# Borderline personality disorder and sexuality: causes and consequences of dissociative symptoms

**DOI:** 10.1186/s40479-024-00251-6

**Published:** 2024-03-19

**Authors:** Rose Gholami Mazinan, Christina Dudek, Hannah Warkentin, Maja Finkenstaedt, Johanna Schröder, Richard Musil, Leonhard Kratzer, Johannes Fuss, Sarah V. Biedermann

**Affiliations:** 1 Social and Emotional Neuroscience Group, Department of Psychiatry and Psychotherapy, Center of Psychosocial Medicine, University Medical Center Hamburg- Eppendorf, Martinistraße 52, 20246 Hamburg, Germany; 2 Department of Psychiatry and Psychotherapy, Psychiatric Clinic of LMU, Munich Ludwig Maximilians-Universität München, München, Germany; 3 Institute for Clinical Psychology and Psychotherapy, Department for Psychology, Medical School Hamburg, Hamburg, Germany; 4 Oberberg Fachklinik Bad Tölz, Bad Tölz, Germany; 5 Department of Psychotraumatology, Clinic St Irmingard, Osternacher Strasse 103, 83209 Prien am Chiemsee, Germany; 6 Institute of Forensic Psychiatry and Sex Research, Center for Translational Neuro- and Behavioral Sciences, University of Duisburg-Essen, Essen, Germany

**Keywords:** Borderline personality disorder, Dissociation, Posttraumatic stress disorder, Child sexual abuse, Sexual risk behavior

## Abstract

**Background:**

Sexual risk behavior in patients diagnosed with borderline personality disorder (BPD) is supposed to be associated with traumatic experiences and dissociative symptoms. Nevertheless, scientific research thereon is scarce which might be due to the high prevalence of sexual trauma and fear of overwhelming patients with explicit sexual content.

**Methods:**

We investigated a clinical sample of patients diagnosed with BPD (*n* = 114) and compared them to a sample of matched healthy controls (HC) (*n* = 114) concerning the dissociative symptoms derealization, depersonalization, and conversion in sexual situations. In a subgroup of patients with BPD (*n* = 41) and matched HC (*n* = 40) dissociative symptoms after exposure to an acoustically presented erotic narrative were assessed in the lab. Regression analyses were used to examine the associations between sexual trauma, post-traumatic stress disorder (PTSD), dissociation in sexual situations, and risky sexual behavior.

**Results:**

Patients diagnosed with BPD endorsed higher dissociative symptoms in sexual situations retrospectively and in the lab compared to HC. Regression analyses revealed that depersonalization and conversion symptoms in sexual situations were explained by severity of BPD, while derealization was explained by PTSD symptomatology. Impulsive and sexual behavior with an uncommitted partner were higher in the BPD group and explained by derealization, while conversion showed an inverse association.

**Conclusion:**

Our findings highlight the importance of addressing distinct dissociative symptoms in sexual situations when counselling and treating women with BPD. In the long term, this could contribute to a reduction in sexual risk behavior in patients with BPD.

**Trial registration:**

This analysis is part of a larger ongoing study and was registered prior to accessing the data (Registration trial DRKS00029716).

**Supplementary Information:**

The online version contains supplementary material available at 10.1186/s40479-024-00251-6.

## Background

Borderline personality disorder (BPD) is a serious mental disorder with an estimated community prevalence of up to 2.7% (Leichsenring et al., 2023). It is characterized by a pervasive pattern of instability in affect, self-image, impulsivity, and interpersonal relationships (American Psychiatric Association, 2013). Problematic and instable intimate relationships as well as self-harming and impulsive sexual behavior have been described as stressful and impairing for patients with BPD and present a strain on their social and intimate relationships [1–3]. However, many aspects of sexual problems associated with BPD have received little scientific attention. A better studied aspect is increased sexual risk, which ranges from lower self-efficacy to refuse sex in youth with BPD [4], higher levels of sexual impulsivity and uncommitted sex [4, 5], increased rates of sexually transmitted diseases, unwanted pregnancies, and commercial sexual relationships [2]. However, underlying mechanisms remain unexplored and many studies were conducted in comorbid substance users [2], thus limiting generalizability.

There is evidence that dissociative symptoms are closely associated with emotional dysregulation and interpersonal problems [6]. Thus, the increased prevalence in patients with BPD [6, 8] and the association with a more complicated course of BPD [7] is not surprising. While competing models and definitions of dissociation exist [4–6], a distinction in symptoms of detachment (e.g. depersonalization and derealization) and compartmentalization (e.g. conversion) is supported by literature [8, 9]. Detachment symptoms, with problems in self-perception (depersonalization) and perception of the environment (derealization), can be understood as a form of protective mechanism in overwhelming situations to create an inner distance, which can be helpful in inescapable threatening situations such as childhood abuse, but can also interfere with safety judgements in social situations [10, 11]. This, in turn, may increase sexual risk through behaviors such as ignoring or downplaying alarm signals due to reduced threat awareness or impaired encoding of threat-related information [10, 11]. Meanwhile, conversion symptoms affect sensory and motor perception of the body and cause somatic dysfunctions, which have no organic basis. This can manifest in the form of paralyzed body parts, paresthesia, and other sensory or motor dysfunctions [12].

Childhood sexual abuse (CSA) is one factor highly prevalent in diagnoses with high dissociative symptoms [13–16] including patients with BPD [17]. Presence of dissociative symptoms in sexual situations might be one way in which sexual risk behavior, but also sexual violence, might be facilitated in the aftermath of having experienced CSA as they could impair awareness and communication of needs and boundaries. This is particularly important given that individuals with BPD are more likely to report sexual revictimization [2]. Associations of revictimization with dissociation in the presence of CSA have indeed been reported in community samples [18, 19].

To the best of our knowledge, the association of dissociation and sexual problems in BPD has received little research attention, especially using experimental methods. This study addresses the associations between sexual stimulation and dissociation using an orally presented erotic narrative as well as retrospective assessments, taking into account the important factors of sexual risk behavior and revictimization, posttraumatic stress disorder (PTSD) and CSA [15, 20]. Due to the lack of experimental sex studies in patients with BPD and the assumed risk that sexual content could trigger distressing traumatic memories in a patient group with a high prevalence of sexual trauma and comorbid PTSD, we used erotic audio stimulation in an experimental setup to create sexual arousal without overwhelming the participants (see methods). Individual reactions were assessed using standardized methods and purpose-designed questionnaires focusing on emotion perception, as well as established parameters of dissociation (derealization, depersonalization and conversion).

We aimed to address the following hypotheses:

1. Patients with BPD report increased dissociative symptoms in sexual situations compared to matched healthy controls.
  - In retrospective analysis using an adapted version of a validated questionnaire.
  - In an experimental sexual arousal induction using an acoustically presented erotic narrative.
2. Dissociative symptoms in sexual situations are influenced by sexual abuse experiences and PTSD symptoms.
3. There is an association between sexual risk behavior and dissociative symptoms.

## Materials and methods

### Study design

Study 1 investigated patients diagnosed with BPD as well as matched healthy controls (HC) using validated and purpose-designed questionnaires while in study 2 we aimed at corroborating our findings in an experimental paradigm. Therefore, we invited a well characterized group of patients with BPD and matched HC in our behavioral laboratory.

### Participants

Patients were recruited at the University Medical Center Hamburg-Eppendorf (UKE) and outpatient clinics in Hamburg, Germany as well as in several in- and outpatient clinics in the Munich region, Germany. HC were recruited through word of mouth and internet advertisements. Inclusion criteria were diagnosed BPD for patients and no current psychiatric disorders for HC. Participants with BPD were excluded from the study if they had acute substance abuse, acute major or delusional depression, mania, or acute psychotic disorders. Due to potential influence of hormonal dysregulation on mood and sexuality in women during menopause, the age range for recruitment was 18 to 45 years. Moreover, we assessed whether participants already had had their menopause, the duration of the menstrual cycle and the first day of their last period.

### Measures

To assess dissociative experiences in sexual situations we used an adjusted version of the well validated questionnaire *Fragebogen zu dissoziativen Symptomen* (FDS; [21, 22]), the German adaptation of the *Dissociative Experience Scale* (DES; [23]), and adapted it to assess dissociative symptoms in typical sexual situations (*FDS*_*Sex*_) by asking: “In a sexual situation that is typical for me…”. This resulted in 16 items assessing dissociative symptoms from 0 to 100% (never to always) when in a typical sexual situation. According to recent developments in the field, additionally to the total score of dissociation, we formed subscores for detachment symptoms (derealization and depersonalization) and conversion as compartmentalization symptoms [24]. While conversion symptoms could be used according to the manual [25], this was not possible for derealization and depersonalization. Thus, we decided to include only two items for the factors derealization *(a. In a sexual situation that is typical for me… - I feel as if I am looking at the world through a veil, so that people and objects seem distant, indistinct, or unreal., b. In a sexual situation that is typical for me… - I have the feeling that other people, objects, and the world around me are not real.)* and depersonalization *(a. In a sexual situation that is typical for me… - I have the feeling that parts of my body change (in size) (e.g. my arms lose their form or become bigger and bigger)., b. In a sexual situation that is typical for me… - I have the feeling that my body or a part of my body does not belong to me)*, which can also be clearly assigned to these phenomena. Internal consistency of this adapted scale ranges from Cronbach’s α = 0.82 to 0.88 for the overall score and the subscales in this sample.

Acute dissociation after the erotic audio was assessed using the German version of the *Dissociation Tension Scale Acute (DSS-acute;* [26]*)*, which is a 22-item questionnaire developed from the DES / FDS (see above) assessing acute dissociation (Cronbach’s α: 0.94).

The *Sexual Risk Survey (SRS)* consists of 23 items assessing various risky sexual behaviors on five categories [27]:

- SRS Uncommitted: Engagement in sexual acts with partners one did not know, trust or were in a relationship with.
- SRS Risky: Sexual risk acts such as unprotected vaginal or oral sex.
- SRS Impulsive: Impulsive and spontaneous sexual acts.
- SRS Intentional: Intentional engagement in risky sex acts.
- SRS Anal: Engagement in risky anal sex.

Each item requires the participant to quantify the number of times they engaged in the questioned sexual behavior in a free-response format. The validated English questionnaire was translated into German, verified with forward-backward-translation (see supplement 4). The timeframe was extended to a 12-month period to be in line with the other measures. Cronbach’s α is ≥ 0.88 for all subscales, except SRS anal with a Cronbach’s α of 0.69.

The subgroup of patients and controls invited into the laboratory were investigated for comorbid diagnoses with the diagnostic short interview for mental disorders (MINI-DIPS; [28]). Diagnosis of BPD was assessed or ruled out according to the Diagnostic and Statistical Manual of Mental Disorders-5 (DSM-5; [29]).

To examine the severity of BPD symptoms in participants, the shortened version of the *Borderline Symptom List (BSL-23)* [30] was applied (Cronbach’s α: 0.94–0.97). The 23 items are answered on a 5-point Likert scale from “not at all” to “very strongly”. The answers refer to the last seven days.

Childhood trauma was assessed with the *Childhood Trauma Questionnaire (CTQ;* [31–33]*)* which consists of 25 clinical and 3 minimizing/denial items, covering the categories of emotional abuse (CEA), physical abuse (CPA), sexual abuse (CSA), emotional neglect (CEN), and physical neglect (CPN) that are answered on a 5-point Likert scale from “never true” to “very often true”. Scores range from 5 to 25 for each type of maltreatment. The CTQ has shown excellent reliability [31, 33] and validity [32–34] with Cronbach’s α of 0.94. Here we used the validated German version of the CTQ [35, 36]. Additionally, a dichotomized variable was created for CSA using a threshold of 7.

Based on the CTQ focusing on traumatic experiences in childhood, we further designed questions that refer to sexual abuse experiences in adulthood (ASA). For this, we adapted four items of the subscale CSA to adulthood (*Adult Sexual Abuse Questionnaire (ASAQ)*, for details see supplement 1).

The *International Trauma Questionnaire (ITQ;* [37]*)* is a diagnostic self-reporting tool designed according to the diagnostic criteria of the 11th version of the International Classification of Diseases (ICD-11) with Cronbach’s α of ≥ 0.77. The 18 items are used to diagnose PTSD and complex PTSD (cPTSD) and are answered on a 5-point Likert scale from “not at all” to “extremely”. Here we used the validated German adaptation [38].

### Procedures

A total of 343 subjects (male = 81; female = 262) between 18 and 45 years of age participated in the study. For study 1 we selected a subgroup of patients and age- and gender-based matched controls, resulting in a final sample of 228 women, 114 in the BPD and 114 in the HC group. Data sets from male participants were excluded for the current analyses due to insufficient sample size. Participants from the control group with a Borderline Symptom List 23 score (BSL-23) of ≥ 2 were also excluded from the analysis due to probability of latent BPD.

All participants gave written informed consent. Participants completed a set of questionnaires from which we selected the German-adapted versions of the above-described questionnaires via the online survey tool Qualtrics®^XM^ and paper pencil for the patients recruited in Munich. The overall response time was about 40 min.

### Experimental exposure to an acoustically presented erotic narrative

The subgroup of participants for study 2 consisted of 41 female participants with a BPD diagnosis and 40 HC. A 5:23-minute-long erotic narrative, which has shown reliable sexual arousal induction [39, 40], was acoustically presented using headphones. A female narrator describes a female-initiated sexual interaction with a man from her point of view. This setup was also beneficial as the female narrated erotic narrative diminished the risk of creating a non-consensual or potentially stressful atmosphere, which could be risked by using a male-initiated erotic narrative, considering the high prevalence of sexual trauma and PTSD in patients with BPD, and also in our patient group (see Table 1).

**Table 1:** Sociodemographic characteristics of the BPD and control group

	Baseline	
	BPD (*n* = 114)	HC (*n* = 114)
**Age in years** ***M (SD)***	28.18 *±* 7.10	28.22 *±* 6.20
**Number of children** ***M (SD)***	1.42 *±* 0.90	1.55 *±* 0.69
**Marital status** ***n (%)***		
Single	56 (49.1)	38 (33.3)
Serious relationship	48 (42.1)	60 (52.6)
Married	3 (2.6)	12 (10.5)
Divorced	2 (1.8)	1 (0.9)
Other	5 (4.4)	3 (2.6)
**Education** ***n (%)***		
No degree	2 (1.8)	1 (0.9)
Low	11 (9.6)	0 (0.0)
Intermediate	45 (39.5)	11 (9.6)
High	50 (43.9)	89 (78.1)
Other	6 (5.3)	13 (11.4)
**Employment status** ***n (%)***		
Full-time employed	16 (14.0))	39 (34.2)
Part-time employed	17 (14.9)	18 (15.8)
Marginally/Occasionally/Irregularly employed	2 (1.8)	6 (5.3)
Unemployed	35 (30.7)	5 (4.4)
Student/In training	28 (24.6)	27 (23.7)
Working Student	10 (8.8))	19 (16.7)
Retired	5 (4.4)	0 (0.0)
Missing Data	1 (0.9)	0 (0.0)
**Sexual orientation** ***n (%)***		
Heterosexual	38 (33.3)	63 (55.3)
Homosexual	4 (3.5)	4 (3.5)
Bisexual	59 (51.8)	45 (39.5)
Pansexual	9 (7.9)	2 (1.8)
Asexual	4 (3.5)	0 (0.0)
**Form of current BPD treatment** ***n (%)***		
Inpatient	44 (38.6)	0 (0.0)
Day clinic	25 (21.9)	0 (0.0)
Outpatient	37 (32.5)	0 (0.0)
None	1 (0.9)	114 (100.0)
Unknown	7 (6.1)	0 (0.0)
**Child sexual abuse** ***n (%)***		
Yes	66 (57.9)	9 (7.9)
No	45 (39.5)	105 (92.1)
Missing data	3 (2.6)	0 (0.0)
**Adult sexual abuse** ***n (%)***		
Yes	51 (44.7)	10 (8.8)
No	53 (46.5)	104 (91.2)
Missing data	10 (8.8)	0 (0.0)
**Revictimization** ***n (%)***		
Yes	41 (36.0)	3 (2.6)
No	60 (52.6)	111 (97.4)
Missing data	13 (11.4)	0 (0.0)
**PTSD** ***n (%)***		
Yes	55 (48.2)	0 (0.0)
No	59 (51.8)	114 (0.0)
Missing data	0 (0.0)	0 (0.0)

*Notes M*: mean; *SD*: standard deviation; *HC*: healthy controls; *BPD*: patients with borderline personality disorder; *PTSD*: posttraumatic stress disorder

Thereafter, all subjects completed the *DSS-acute*. Out of six subscales, we here used derealization, depersonalization, and conversion. Each item can be rated on a 10-point Likert scale from “0 = sensation was not observed” to “10 = sensation was very strong”. Additionally, participants indicated the degree to which sexual arousal, tension, and a selection of aversive (shame, anger, fear, disgust) and affirmative emotions (curiosity, sexual desire) were experienced during the erotic narrative on a scale from 0 to 100%. All experiments were conducted in one session in a separate test room with the experimenter immediately available upon request.

## Statistical analysis

Sociodemographic sample characteristics were compared between patients and HC using student’s t-test and chi-square as appropriate. We then conducted further analyses to examine the associations between experiences of sexual abuse, PTSD symptomatology, and self-reported sexual risk behaviors, with dissociation in sexual situations in BPD.

Two-sample student’s t-test was used to examine the degree of dissociation including the subscores derealization, depersonalization, and conversion in sexual situations retrospectively and after the erotic narrative comparing patients with BPD and HC. Moreover, intensity of the emotions and tension after the audio were exploratively correlated with overall dissociation and the dissociation subscores using Pearson’s correlation coefficient. Furthermore, student’s t-test was used to compare the subscores of the SRS, CTQ, and ASAQ between patients with BPD and HC. Revictimization was defined as ASAQ and CSA subscore of the CTQ being simultaneously present (cut off = 7). Further analyses were carried out using chi-square-test.

To investigate the associations of these factors with dissociative symptoms, correlation analyses using Pearson correlation coefficient were conducted. Mann-Whitney-U-tests were calculated to examine if presence of revictimization influenced dissociative symptoms in sexual situations in the patient sample.

Thereafter, two sets of regression analyses using backward elimination were calculated in the BPD group only: (i) to examine the influence of clinical characteristics on dissociation for the three subscores of the FDS_Sex_ as well as the total score with the independent variables CSA, ASA, BSL-23, and ITQ-PTSD sumscore, and (ii) to examine the influence of dissociation and other clinical characteristics on sexual risk behavior with the independent variables CSA, BSL-23, ITQ-PTSD sumscore, and the three subscores of the FDS_Sex_.

All statistical tests were performed using IBM SPSS ® version 27. Statistical significance was assumed at *p* <.05. Cohen’s d was used to report effect size.

## Results

### Sociodemographic characteristics

There were no significant differences concerning age, number of children, marital status, employment status and sexual orientation between HC and patients with BPD (see Table 1). However, the frequency of PTSD (t(161.07) = 13.31; p = < 0.001; d = 1.76), sexual abuse experiences in childhood (t(116.64) = 9.58; p = < 0.001; d = 1.29), adulthood (t(128.59) = 7.59; p = < 0.001; d = 1.07), revictimization (x^2^ [1] = 47.42; p = < 0.001; ϕ = <0.001) as well as all CTQ subscores (all *p* <.001, for details see Supplement 3) were higher in the patient group.

#### Dissociation in sexual situations retrospectively and in the lab

##### Retrospective analysis of dissociation in sexual situations

T-test revealed higher levels of total dissociation in sexual situations in patients compared to HC (t(146.54) = 3.75; p = < 0.001; d = 0.50). More precisely, patients with BPD reported significantly higher derealization (t(226) = 3.419; p = < 0.001; d = 0.45), depersonalization (t(226) = 4.01; *p* <.001; d = 0.53) and conversion (t(167.90) = 2.19; *p* =.030; d = 0.29), in sexual situations (see Table 2).

**Table 2:** FDS_Sex_ scores (M/SD): Group difference in total dissociation, derealization, depersonalization, and conversion in sexual situations

	BPD (*n* = 114)	HC (*n* = 114)	*p*	Effect size
FDS_Sex_ total dissociation	3.95 ± 6.81	1.38 ± 2.65	< 0.001**	d = 0.50
FDS_Sex_ derealization	6.47 ± 12.54	1.76 ± 5.33	< 0.001**	d = 0.45
FDS_Sex_ depersonalization	4.54 ± 8.41	1.20 ± 2.94	< 0.001**	d = 0.53
FDS_Sex_ conversion	2.58 ± 5.84	1.24 ± 2.98	0.030*	d = 0.29

*Notes M*: mean; *SD*: standard deviation; *HC*: healthy controls; *BPD*: patients with borderline personality disorder; *FDS*_*Sex*_: Fragebogen zu dissoziativen Symptomen (während des Sex) /Dissociative symptoms questionnaire (during sex); *p < 0.05 and **p <.001 indicate statistically significant differences between groups; *d*: Cohen’s d

##### Exposure to an erotic narrative in the lab

Acute total dissociation (t(79) = 2.41; *p* =.018; d = 0.54), derealization (t(59.20) = 2.51; *p* =.015; d = 0.55), and depersonalization (t(62.31) = 2.90; *p* =.005; d = 0.64), were significantly higher in patients than in HC after exposure to an erotic narrative in the lab (see Table 3). Conversion (t(79) = 1.72; *p* =.089; d = 0.38) was more frequent in patients on a descriptive level not reaching significance (see Table 3).

**Table 3:** DSS-acute scores (M/SD): Group difference in acute dissociation, derealization, depersonalization, and conversion after erotic narrative

	BPD (*n* = 41)	HC (*n* = 40)	*p*	Effect size
DSS total dissociation	1.10 ± 1.26	0.54 ± 0.77	0.018*	d = 0.54
DSS derealization	0.68 ± 1.10	0.19 ± 0.55	0.015*	d = 0.55
DSS depersonalization	1.24 ± 1.48	0.48 ± 0.81	0.005*	d = 0.64
DSS conversion	9.02 ± 11.75	5.15 ± 8.08	0.089	d = 0.38

*Notes M*: mean; *SD*: standard deviation; *HC*: healthy controls; *BPD*: patients with borderline personality disorder; *DSS*: dissociation tension scale acute; *p < 0.05 indicates statistically significant differences between groups;*d*: Cohen’s d

##### Emotions during the erotic narrative

Sexual arousal was slightly higher in HC, tension during and after the erotic narrative was slightly higher in patients without any significant differences in any of the emotions assessed (see Table 4). In HC, weak correlations between shame and dissociative symptoms (total dissociation: *r* =.32; *p* =.047; derealization: *r* =.37; *p* =.019; conversion: *r* =.33; *p* =.036) as well as between sexual arousal and dissociation (total dissociation: *r* =.35; *p* =.029; conversion: *r* =.40; *p* =.010) were found. In the BPD group, weak to moderate correlations of fear (total dissociation: *r* =.49; *p* =.001; derealization: *r* =.41; *p* =.009; depersonalization: *r* =.37; *p* =.019; conversion: *r* =.48; *p* =.002) and curiosity (total dissociation: *r* =.54; p = < 0.001; derealization: *r* =.35; *p* =.028; depersonalization: *r* =.37; *p* =.019; conversion: *r* =.61; p = < 0.001) with dissociation were seen. Conversion showed a moderate correlation with curiosity (*r* =.61; p = < 0.001). Sexual arousal correlated with total dissociation (*r* =.44; *p* =.004) and conversion (*r* =.43; *p* =.006). Moreover, tension during and after the audio correlated positively with various dissociative symptoms with a small effect size (see supplement 2).

**Table 4:** Group difference in sexual arousal, tension, and emotions during the erotic narrative (M/SD)

	BPD (*n* = 41)	HC (*n* = 40)	*p*	Effect size
Sexual Arousal during Audio	32.37 ± 29.46	34.08 ± 27.38	0.788	d = -0.06
Tension during Audio	26.17 ± 29.73	20.48 ± 29.67	0.391	d = 0.19
Tension after Audio	2.34 ± 2.29	1.68 ± 2.46	0.211	d = 0.28
Shame^a^	27.55 ± 28.86	23.45 ± 32.40	0.552	d = 0.13
Anger^a^	2.13 ± 7.22	4.50 ± 16.85	0.415	d = -0.18
Fear^a^	3.48 ± 11.94	3.83 ± 16.30	0.913	d = -0.03
Disgust^a^	16.18 ± 22.69	18.73 ± 30.09	0.670	d = -0.10
Curiosity^a^	38.30 ± 29.26	41.18 ± 33.04	0.681	d = -0.09
Sexual desire^a^	34.75 ± 31.19	37.35 ± 29.09	0.701	d = -0.09

*Notes M*: mean; *SD*: standard deviation; *HC*: healthy controls; *BPD*: patients with borderline personality disorder; *d*: Cohen’s d; a: missing patient data *n* = 1

#### Influence of sexual abuse and PTSD symptoms on dissociative experiences in sexual situations

We analyzed the associations between (i) childhood sexual abuse, (ii) adult sexual abuse, (iii) revictimization and (iv) PTSD with dissociative experiences in sexual situations in the patient group only using the FDS_sex_:

I. Intensity of CSA correlated weakly with depersonalization (*r* =.21; *p* =.029). Patients with BPD and endorsed CSA did not report significantly more dissociation in sexual situations than patients without.
II. Weak positive correlations for severity of ASA with total dissociation (*r* =.19; *p* =.049) and conversion (*r* =.23; *p* =.019), but not derealization and depersonalization, were found. Patients with BPD, who endorsed ASA, did not report more dissociative symptoms in sexual situations than patients without.
III. Revictimization was associated with more dissociative symptoms in sexual situations (total dissociation: U = 1,647.00; Z = 2.89; *p* =.004; derealization: U = 1,699.50; Z = 3.254; *p* =.001; conversion: U = 1,638.00; Z = 2.90; *p* =.004), except for depersonalization (U = 1,374.00; Z = 124; *p* =.217).
IV. Patients with BPD fulfilling criteria for PTSD in the ITQ experienced significantly higher levels of dissociation (total dissociation: t(63.61) = 3.45; *p* =.001; d = 0.67; derealization: t(68.57) = 3.22; *p* =.003; d = 0.60; depersonalization: t(85.76) = 3.139; *p* =.002; d = 0.60; conversion: t(61.89) = 2.52; *p* =.014; d = 0.49) than those without PTSD. Furthermore, total dissociation (*r* =.35; *p* <.001), derealization (*r* =.30; *p* =.001), depersonalization (*r* =.28; *p* =.002), and conversion (*r* =.27; *p* =.003) correlated with PTSD total score.

Linear regression analyses revealed a significant effect of the intensity of BPD symptoms (BSL) on total dissociation (ß = 0.377; t = 3.899; *p* <.001), conversion (ß = 0.321; t = 3.254; *p* =.002), and depersonalization (ß = 0.305; t = 3.074; *p* =.003). Meanwhile, derealization was best explained by PTSD symptomatology (ß = 0.291; t = 2.922; *p* =.004). The other factors did not have a significant effect in the regression analyses.

#### Association of dissociation and sexual risk behavior

Only two out of five subscores of the SRS, SRS Impulsive (t(130.86) = 3.25; *p* =.001; d = 0.46) and SRS Uncommitted (t(111.71) = 2.38; *p* =.019; d = 0.34), were significantly different between patients with BPD and HC (see Table 5).

**Table 5:** Sexual risk behavior (SRS-scores (M/SD)) in women with and without BPD

	BPD (*n* = 114) ^a^	HC (*n* = 114)	*p*	Effect size
SRS Uncommitted	32.70 ± 89.58	11.37 ± 19.28	0.019*	d = 0.34
SRS Risky	84.75 ± 159.14	79.61 ± 101.11	0.774	d = 0.04
SRS Impulsive	11.05 ± 17.63	5.05 ± 6.82	0.001*	d = 0.46
SRS Intentional	2.46 ± 7.11	2.06 ± 6.24	0.664	d = 0.06
SRS Anal	23.94 ± 144.99	4.55 ± 18.23	0.179	d = 0.19

*Notes M*: mean; *SD*: standard deviation; *HC*: healthy controls; *BPD*: patients with borderline personality disorder; *SRS*: sexual risk survey; *p < 0.05 indicates statistically significant differences between groups; *d*: Cohen’s d; *a*: missing patient data *n* = 10

Linear regression analyses in the BPD group only showed that SRS Uncommitted was explained by derealization in the FDS_Sex_ (ß = 0.335; t = 3.519; *p* <.001). SRS Impulsive was explained by derealization (ß = 0.573; t = 4.891; *p* <.001) and conversion (ß = − 0.464; t = -3.981; *p* <.001) in diverging directions.

## Discussion

The current study aimed to shed light on the underpinning factors and possible effects of dissociative experiences in sexual situations investigating patients with BPD. Research explicitly focusing on dissociation in BPD is relatively scarce and this is even more the case for dissociation in sexual situations [6]. Firstly, our study provides evidence that experimental sex research in a laboratory is well tolerated and feasible among patients with BPD, at least with a trained research team and necessary clinical expertise.

Our main finding is that people with BPD reported higher levels of dissociation in sexual situations compared to matched controls, both, in a retrospective assessment as well as under laboratory conditions. Effects were more pronounced for derealization and depersonalization than for conversion. Depersonalization in sexual situations was mainly explained by severity of BPD-symptomatology while derealization in sexual situations was mainly explained by PTSD-symptomatology. Meanwhile, higher likelihood of sexual behavior with an uncommitted sex partner was mainly explained by derealization in sexual situations, while impulsive sexual behavior was explained by derealization and conversion in diverging directions.

The concept of dissociation is only partially understood, and we have attempted here to address the distinct dissociative symptoms of derealization, depersonalization and conversion. We used an adapted version of the DES [25], to assess dissociative symptoms in sexual situations. Although the DES is one of the best established measures of dissociative symptoms, it is also the oldest, reflecting an outdated concept of dissociations [41, 42], while competing models and definitions of dissociation exist [8, 43, 44]. We have opted here for a multifaceted approach that focuses on phenomenology and distinguishes between detachment (derealization and depersonalization) and compartmentalization (conversion) [8, 9, 45], which can be assessed using the FDS / DES [24].

### Exposure to an erotic narrative

Even though patients with BPD endorsed significantly higher levels of dissociative symptoms after listening to the erotic narrative, there were no significant differences regarding intensity of any of the emotions assessed. This is in contrast to at least some former studies that found increased levels of emotional reactivity in patients with BPD [46–48] and this also corresponded to diminished amygdala habituation [49]. Meanwhile, the positive association between dissociative experiences and sexual arousal in both groups indicates that dissociative symptoms associated with sex are within the range of physiological reactions, at least when tested under laboratory conditions. At the same time, many emotions assessed including aversive tension, but not shame, anger, and disgust, positively correlated with dissociative experiences after the erotic narrative in the BPD group. Aversive tension is described as a state of uncomfortable high arousal which is perceived subjectively by the individual [50, 51]. It has been associated with dissociative symptoms in BPD before [52], especially if exposed to psychological stressors [53] while dissociation may function as one mechanism to escape from tense, stressful situations [54]. The fact that intensity of perception of many emotions, including curiosity, sexual desire and arousal, correlated with dissociative symptoms in the BPD group, indicates a rather generalized perception of emotions as stressful and unpleasant, possibly adding to the hypothesis that patients with BPD tend to avoid intense emotions altogether [55]. In this regard, the absence of a correlation between perception of shame and anger with dissociation could also hint to an avoidance mechanism of these emotions in sexual situations. Meanwhile, besides sexual arousal, only the emotion shame was associated with dissociative symptoms in the control group, suggesting a dominantly negative association of dissociation with aversion and discomfort in controls.

Patients reported increased symptoms of detachment compared to the control group, namely derealization and depersonalization, in the retrospective analysis as well as during the erotic audio stimulation. Meanwhile, there was a smaller effect on conversion symptoms with no significant difference between the groups in the laboratory setting. Interestingly, a former questionnaire study found an association of fewer problems with sexual arousal and higher derealization symptoms in women with and without endorsement of CSA, while in the control group, depersonalization was associated with lower sexual arousal [56]. This might indicate that certain dissociative symptoms have distinct associations with sexual function irrespective of a history of sexual abuse, which should be studied more in depth in the future.

### Influence of abuse and trauma on dissociation in BPD

Even though it is difficult to disentangle the effects of CSA and PTSD on dissociative symptoms in sexual situations from one another due to the broad overlap, we aimed to include these factors in the current analyses. In contrast to former studies that reported increased level of dissociation in sexual situations in participants endorsing CSA compared to control participants [56, 57] we could not replicate this finding. One reason might be that the aforementioned studies did not take the factor of possible BPD pathology into account. Moreover, even though we could corroborate the finding of increased endorsement of ASA in BPD [17], this was not associated with higher dissociative experience in sexual situations.

However, women with BPD, who endorsed sexual revictimization reported significantly more dissociative symptoms in sexual situations, which is in line with earlier studies that reported associations between dissociation and an increased risk for revictimization [18, 19].

Moreover, about half of the BPD group fulfilled criteria for PTSD, which often is associated with dissociative symptoms [15, 20, 58, 59]. Thus, our finding of a significant association of PTSD symptoms with all FDS_Sex_ items at the group level and at the correlational level is not surprising. Even though these correlative associations and group differences suggest an influence of PTSD on dissociative experiences in sexual situations in BPD, our regression analyses in the patient group showed otherwise. Interestingly, we found in the regression analyses that depersonalization and conversion in sexual situation were best explained by intensity of borderline symptoms (BSL-23), while derealization was best explained by PTSD-symptomatology. This suggests that the underlying mechanisms for dissociative symptoms in sexual situations, at least in patients with BPD, go beyond the experience of sexual trauma and should be better investigated taking the factors of BPD- and PTSD-symptoms into account.

### Is dissociation in BPD associated with sexual risk behavior?

We could corroborate the finding that patients with BPD more often engage in impulsive and uncommitted sex [4, 5, 60]. We complement these findings by showing that participants who reported higher levels of derealization were also more likely to engage in impulsive and uncommitted sex, whereas an inverse relationship was found for conversion in relation to impulsive sexual behavior.

The finding is in line with a recent study in an online convenience sample, where the number of BPD symptoms correlated with dissociative symptoms in daily life, and this was associated with increased risk of being a victim of intimate partner violence [6]. Meanwhile, the clinical significance of conversion on sexual symptomatology awaits further investigation even though one could speculate that conversion symptoms in sexual situations might promote aversion of sexual situations and somewhat function as a protective factor.

It was reported before that CSA might be a risk factor for more frequent sexual risk behavior [61, 62] and also with sexual preferences causing distress [63]. Our regression analyses could not support a strong direct association of CSA with sexual risk behavior. However, with a more homogenous and bigger sample possible moderating effects of traumatic experiences on dissociation and risk behavior in sexual situations as well as sexual preferences causing distress should be examined.

### Limitations

Due to the inclusion of female subjects only, we can only draw conclusions on female patients with BPD. Since patients with a high interest in sexuality and present sexual difficulties were more willing to participate and patients with severe PTSD were more likely to withdraw due to fear of experiencing PTSD symptoms, a recruitment bias could limit the generalizability of our results. Moreover, differences in therapeutic experiences of the participating patients might have influenced our results.

## Conclusion

We conclude that experimental sex research can be performed safely in patients with BPD under controlled conditions. Increased level of depersonalization and conversion in sexual situations were best explained by BPD symptoms while derealization seemed to be mainly influenced by PTSD-symptoms in patients with BPD. Meanwhile, higher impulsive and uncommitted sexual behavior in BPD were best explained by intensity of derealization during sexual situations, while conversion might somehow function as protective factor.

Given the diverging effects of derealization and conversion on sexual risk behavior, a distinct assessment of dissociative experiences in patients with BPD should be performed in clinical and research settings. The influence of PTSD symptoms is particularly worth exploring in this context. In a therapeutic context, it might be worth to specifically address derealization in sexual situations to reduce sexual risk behavior.

### Electronic supplementary material

Below is the link to the electronic supplementary material.

Supplementary Material 1

## Data Availability

No datasets were generated or analysed during the current study.

## Abbreviations

ASA: Adult Sexual Abuse
ASAQ: Adult Sexual Abuse Questionnaire
BSL-23: Borderline Symptom List 23
CEA: Childhood Emotional Abuse
CEN: Childhood Emotional Neglect
CPA: Childhood Physical Abuse
CPN: Childhood Physical Neglect
CPTSD: Complex Post-Traumatic Stress Disorder
CSA: Childhood Sexual Abuse
CTQ: Childhood Trauma Questionnaire
DES: Dissociative Experience Scale
DSM-5: Diagnostic and Statistical Manual of Mental Disorders 5
DSS-acute: Dissociation Tension Scale Acute
FDS: Fragebogen zu dissoziativen Symptomen/ Dissociative Symptoms Questionnaire
FDSsex: Fragebogen zu dissoziativen Symptomen Sex/ Dissociative Symptoms during Sexual Activity Questionnaire
HC: Healthy controls
ICD-11: International Classification of Diseases 11th Version
ITQ: International Trauma Questionnaire
LMU: Ludwig-Maximillians-University Munich
MINI-DIPS: Diagnostic Short Interview for Mental Disorders
PTSD: Post-Traumatic Stress Disorder
SRS: Sexual Risk Survey
UKE: University Medical Center Hamburg-Eppendorf
