# Supplementary material for: Borderline personality disorder and sexuality: causes and consequences of dissociative symptoms
Source: Borderline Personal Disord Emot Dysregul. 2024 Mar 19;11:8. doi: 10.1186/s40479-024-00251-6 (PMC10949637; doi:10.1186/s40479-024-00251-6)
Supplement: Supplementary file 1 — Supplementary Material 1 [file 40479_2024_251_MOESM1_ESM.docx]

**Supplement 1.** Childhood sexual abuse score of the CTQ modified for adulthood (Adult sexual abuse questionnaire, ASAQ)

The Adult Sexual Abuse Questionnaire (ASAQ) consists of four questions:

- As an adult... - someone threatened to hurt me or tell lies about me if I did not perform sexual acts with him or her.
- As an adult... - someone tried to get me to do or watch sexual things.
- As an adult... - someone sexually harassed me.
- As an adult... - I was sexually abused.

These questions are answered on a 5-point Likert scale from “never true” to “very often true”.

**Supplement 2.** *Arousal induction with* *erotic narrative and DSS-acute scores: influence of sexual arousal, tension and different distinct emotions on acute dissociative experience in women with and without BPD.*

| **BPD (*n* = 41)** | | **DSS**  **dissociation** | **DSS**  **derealization** | **DSS**  **depersonalization** | **DSS**  **conversion** |
| --- | --- | --- | --- | --- | --- |
| **Sexual arousal during audio** | r | 0.44 | 0.21 | 0.25 | 0.43 |
|  | p | **.004** | .188 | .121 | **.006** |
| **Audio tension** | r | 0.35 | 0.37 | 0.28 | 0.37 |
|  | p | **.025** | **.018** | .080 | **.018** |
| **Post-audio tension** | r  p | 0.30  **.058** | 0.39  **.012** | 0.26  .098 | 0.23  .153 |
| **Shame^a^** | r | 0.15 | 0.20 | 0.24 | 0.04 |
|  | p | .370 | .226 | .129 | .824 |
| **Anger^a^** | r | 0.21 | 0.31 | 0.29 | 0.18 |
|  | p | .186 | **.048** | .074 | .263 |
| **Fear^a^** | r | 0.49 | 0.41 | 0.37 | 0.48 |
|  | p | **.001** | **.009** | **.019** | **.002** |
| **Disgust^a^** | r | 0.06 | -0.03 | 0.22 | -0.02 |
|  | p | .735 | .875 | .182 | .919 |
| **Curiosity^a^** | r | 0.54 | 0.35 | 0.37 | 0.61 |
|  | p | **<.001** | **.028** | **.019** | **<.001** |
| **Sexual desire^a^** | r | 0.36 | 0.14 | 0.25 | 0.35 |
|  | p | **.024** | .375 | .128 | **.026** |
| **HC (*n* = 40)** |  |  |  |  |  |
| **Sexual arousal during audio** | r | 0.35 | 0.19 | 0.20 | 0.40 |
|  | p | **.029** | .233 | .233 | **.010** |
| **Audio tension** | r | 0.12 | 0.25 | 0.08 | 0.17 |
|  | p | .481 | .123 | .607 | .296 |
| **Post-audio tension** | r  p | 0.27  .098 | 0.40  **.010** | 0.28  .080 | 0.28  .077 |
| **Shame** | r | 0.32 | 0.37 | 0.20 | 0.33 |
|  | p | **.047** | **.019** | .211 | .**036** |
| **Anger** | r | -0.08 | -0.04 | -0.06 | -0.06 |
|  | p | .634 | .821 | .717 | .729 |
| **Fear** | r | -0.00 | 0.05 | -0.00 | 0.02 |
|  | p | .980 | .781 | .989 | .897 |
| **Disgust** | r | -0.14 | -0.06 | -0.18 | -0.10 |
|  | p | .400 | .693 | .281 | .532 |
| **Curiosity** | r | 0.10 | -0.04 | -0.13 | 0.15 |
|  | p | .528 | .796 | .419 | .361 |
| **Sexual desire** | r | 0.23 | 0.09 | 0.06 | 0.27 |
|  | p | .153 | .583 | .707 | .090 |

Notes. HC: healthy controls. BPD: patients with borderline personality disorder. DSS-acute: dissociation tension scale acute. r: Pearson correlation. a: missing patient data n = 1.

**Supplement 3.** *CTQ, ASA scores (M/SD): different forms of childhood trauma and adult sexual abuse in women with and without BPD.*

|  | BPD  (n = 114) | HC  (n = 114) | p | Effect size | |
| --- | --- | --- | --- | --- | --- |
| CTQ total score  CTQ sexual abuse  CTQ emotional abuse  CTQ emotional neglect  CTQ physical abuse  CTQ physical neglect  ASAQ total score | 64.26 ± 20.76^a^  11.26 ± 6.32^b^  17.70 ± 5.71^c^  16.70 ± 5.71  8.76 ± 4.11  10.26 ± 4.04^d^  8.55 ± 4.35^e^ | 35.46 ± 9.82  5.43 ± 1.11  8.25 ± 3.76  9.46 ± 4.54  5.52 ± 1.66  6.82 ± 2.55  5.11 ± 1.61 | **<.001**  **<.001**  **<.001**  **<.001**  **<.001**  **<.001**  **<.001** | | d = 1.79  d = 1.29  d = 1.96  d = 1.41  d = 1.04  d = 1.02  d = 1.07 |

Notes. M: mean. SD: standard deviation. HC: healthy controls. BPD: patients with borderline personality disorder. CTQ: child trauma questionnaire. ASAQ: adult sexual abuse questionnaire. d: Cohen’s d. a: missing patient data n = 6. b: missing patient data n = 3. c: missing patient data n = 1. d: missing patient data n = 2. e: missing patient data n = 10.

**Supplements 4.** *Sexual risk score in English original and German forward-backward translation.*

**Fragebogen zu sexuellem Risikoverhalten (SRS)**

***Questionnaire on sexual risk behaviour***

Bitte lesen Sie sich die folgenden Aussagen durch und tragen Sie die für Sie richtige Zahl, in Bezug auf die **letzten 6 Monate** in das jeweilige Kästchen ein. Wenn Sie nicht genau wissen, wie häufig ein Verhalten stattgefunden hat, versuchen Sie Häufigkeit möglichst genau zu schätzen. Es kann leichter sein, wenn Sie an die durchschnittliche Häufigkeit denken, an denen das Verhalten pro Woche oder pro Monat stattgefunden hat, v.a. dann, wenn das Verhalten ziemlich regelmäßig stattgefunden hat. Wenn Sie mehrere Partner/Partnerinnen hatten, versuchen Sie zu überlegen, wie lange Sie mit jedem Partner/jeder Partnerin zusammen waren, wie viele sexuelle Begegnungen Sie jeweils hatten und versuchen Sie eine genaue Schätzung der Gesamtzahl von jedem Verhalten zu erzielen. Wenn die Frage nicht auf Sie zutrifft oder Sie das Verhalten in der Frage nie durchgeführt haben, tragen Sie eine „0“ in das Kästchen ein. Bitte lassen Sie keine Items frei.

*Please read the following statements and enter the appropriate number for you in the respective answer box, based on the last 6 months. If you are not exactly sure how often a type of behaviour has occurred, try and estimate the frequency, as accurately as possible. It could be easier to think about the average frequency of the behaviour per week or per month particularly if the behaviour has happened quite frequently. If you had several partners, try to think about how long you were with each partner for, how many sexual encounters you had in each case, and try to provide an accurate estimate of the total occurrences of each behaviour. If the question is not relevamt for you or you have never participated in the behaviour referenced in the question, enter a 0 in the answer box. Please don't leave any items unanswered.*

Beachten Sie, dass „Sex“ bei den folgenden Fragen „Oral-, Anal- und Vaginalsex“ umfasst und „Sexuelle Aktivität“ „leidenschaftliches Küssen, Herummachen, Fummeln, Vorspiel, Petting, anale Stimulation mit dem Mund sowie genitale Stimulation mit der Hand“ einschließt. Wörter, bei denen Sie sich nicht sicher sind, finden Sie in dem Glossar. Bitte berücksichtigen Sie bei Ihren Angaben nur die letzten **6 Monate** und seien Sie bitte ehrlich.

*Note that sex for the purpose of the following questions extends to oral, anal and vaginal sex and secual activity includes passionate kissing, making out, touching, foreplay, petting, anal stimulation with the mouth and manual genital stimulation.  Words which may be unclear are defined in the glossary. Please take into account only the last six months and please be honest.*

| In den letzten 6 Monaten…  *In the last 6 months…* |
| --- |
| 1. Mit wie vielen Partnern hatten Sie Sie sexuelle Aktivitäten, ohne Sex zu haben?/ *How many partners did you engage in sexual activity with without having sex?* |
| 1. Wie häufig haben Sie eine soziale Veranstaltung mit jemandem verlassen, den Sie gerade kennengelernt haben? / *How often did you leave a social event with someone who you only just met?* |
| 1. Wie häufig haben Sie jemanden „aufgerissen/abgeschleppt“, den/die Sie nicht oder nicht gut kannten, ohne Sex zu haben? / *How often did you hook up with someone who you didnt know well without having sex?* |
| 1. Wie häufig sind Sie in Bars/auf Partys/soziale Veranstaltungen gegangen, mit der Absicht, jemanden „aufzureißen/abzuschleppen“ und sich auf sexuelle Aktivitäten einzulassen, aber keinen Sex zu haben? / *How often did you go to bars, parties, social events with the intention of hooking up with someone and engaging in sexual activity, but without having sex?* |
| 1. Wie häufig sind Sie in Bars/auf Partys/soziale Veranstaltungen gegangen, mit der Absicht, jemanden „aufzureißen/abzuschleppen“ und Sex zu haben? / *How often did you go to bars, parties, social events with the intention of hooking up with someone and having sex?* |
| 1. Wie häufig hatten Sie ein unerwartetes und unvorhergesehenes sexuelles Erlebnis? / *How often did you have an unanticipated/unplanned sexual encounter?* |
| 1. Wie häufig hatten Sie eine sexuelle Begegnung mit jemandem, auf die Sie sich willentlich eingelassen, aber später bereut haben? / *How often did you have a sexual encounter with someone which you voluntarily participated in, but later regretted?* |

Bitte beantworten Sie die nächste Reihe an Fragen genauso wie davor. Wenn Sie jedoch hinsichtlich der nächsten Fragen (8-23) keinen Sex hatten (oral, anal oder vaginal), tragen Sie bitte eine „0“ in jedes Kästchen ein.

*Please answer the next series of questions in the same way. For the next questions, if you didn‘t have sex (oral, anal or vaginal) please enter a 0 in each answer box.*

| In den letzten 6 Monaten …  *In the last 6 months…* |
| --- |
| 1. Mit wie vielen Partnern hatten Sie Sex? / *How many partners did you have sex with?* |
| 1. Wie häufig hatten Sie Vaginalsex ohne ein Latex- oder Polyurethan-Kondom? Hinweis: Schließen Sie die Male ein, bei denen Sie ein Lammhaut- oder Membran-Kondom verwendet haben. / *How often did you have vaginal sex without a latex or polyurethane condom? Note: include occasions where you used a lambskin or rubber condom.* |
| 1. Wie häufig hatten Sie Vaginalsex ohne Schutz vor einer Schwangerschaft? / *How often did you have vaginal sex without using contraception to prevent pregancy?* |
| 1. Wie häufig haben Sie Fellatio (Oralsex beim Mann; „Blow Job“) ohne Kondom durchgeführt oder selbst erhalten? / *How often did you give or receive fellatio (oral sex with a male “blow job”) wihtout using a condom?* |
| 1. Wie häufig haben Sie Cunnilingus (Oralsex bei einer Frau, „Lecken“) ohne ein Dental Dam oder „adäquaten Schutz“ durchgeführt oder selbst erhalten? (Bitte Definition von Dental Dam in der Fußnote beachten, um zu sehen, was als adäquater Schutz betrachtet wird). / *How often did you give or receive cunnilingus (oral sex with a woman, “going down”) without a dental dam or adequate protection? (please see definition of dental dam in the footnote to see what is considered adequate protection)* |
| 1. Wie häufig hatten Sie Analsex ohne ein Kondom? / *How often did you have anal sex without a condom?* |
| 1. Wie häufig haben Sie oder Ihr Partner/Ihre Partnerin Analsex mit der Hand („Fisting“) oder einem anderen Objekt ohne einen Latexhandschuh oder ein Kondom durchgeführt, gefolgt von ungeschütztem Analsex? / *How often did you or your partner have manual anal sex (“fisting”) without a latex glove or condom followed by unprotected anal sex?* |
| 1. Wie häufig haben Sie Analingus (= orale Stimulation der Analregion mit der Zunge, „Rimming“) ohne ein Dental Dam^1^ oder „adäquaten Schutz“ durchgeführt oder selbst erhalten? (Bitte Definition von Dental Dam in der Fußnote beachten, um zu sehen, was als adäquater Schutz betrachtet wird). / *How often did you give or receive analingus (oral stimulation of the anal region with the tongue "Rimming") without a dental dam or adequate protection? (please see definition of dental dam in the footnote to see what is considered adequate protection)* |
| 1. Mit wie vielen Personen, die Sie kennen, aber mit denen Sie nicht in irgendeiner Art von Beziehung stehen (d.h. „Freunde mit Vorzügen“, „Fickfreunde“), hatten Sie Sex? *With how many people that you know, but with whom you have no form of relationship, have you had sex (i.e. “friends with benefits”, “fuck buddies”)?* |
| 1. Wie häufig hatten Sie Sex mit jemandem, den/die Sie nicht gut kennen oder den/die Sie gerade kennengelernt haben? */ How often did you have sex with someone who you didn’t know well or only just met?* |
| 1. Wie häufig haben Sie oder Ihr/e Partner/in vor oder während des Sex‘ Alkohol oder Drogen konsumiert? / *How often did you or your partner consume alcohol or drugs before or during sex?* |
| 1. Wie häufig hatten Sie Sex mit einem neuen Partner/einer neuen Partnerin, bevor Sie über die sexuelle Vorgeschichte, intravenösen Drogenkonsum, Krankheitsstatus und andere aktuelle Sexualpartner gesprochen haben? / *How often did you have sex with a new partner without discussing sexual history, intravenous drug consumption, diseases and other current sexual partners?* |
| 1. Wie viele Male (von denen Sie wissen) hatten Sie Sex mit jemandem, der viele Sexualpartner hatte? / *How often (to your knowledge) have you had sex with someone who had many sexual partners?* |
| 1. Mit wie vielen Partnern (von denen Sie wissen) hatten Sie Sex, die sexuell aktiv waren, bevor Sie mit Ihnen zusammen waren, die aber nicht auf sexuell übertragbare Krankheiten/HIV getestet wurden? / *How many partners (to your knowledge) have you had sex with who were sexually active before you were together but hadn’t been tested for sexually transmitted diseases or HIV?* |
| 1. Mit wie vielen Partnern hatten Sie Sex, denen Sie nicht vertrauten? / *How many people have you had sex with who you didn‘t trust?* |
| 1. Wie viele Male (von denen Sie wissen) hatten Sie Sex mit jemandem, der/die im selben Zeitraum auch Sex mit anderen hatte? / *How many times (to your knowledge) did you have sex with someone who was also having sex with someone else at the same time?* |
